# Supplementary material for: The amyloid structure of mouse RIPK3 (receptor interacting protein kinase 3) in cell necroptosis
Source: Nat Commun. 2021 Mar 12;12:1627. doi: 10.1038/s41467-021-21881-2 (PMC7955032; doi:10.1038/s41467-021-21881-2)
Supplement: Supplementary file 1 — Supplementary Information [file 41467_2021_21881_MOESM1_ESM.pdf]

Supplementary Information

The Amyloid Structure of Mouse RIPK3 (Receptor Interacting Protein Kinase 3) in Cell Necroptosis

Xia-lian Wu, Hong Hu et al.

Supplementary Information

a

b

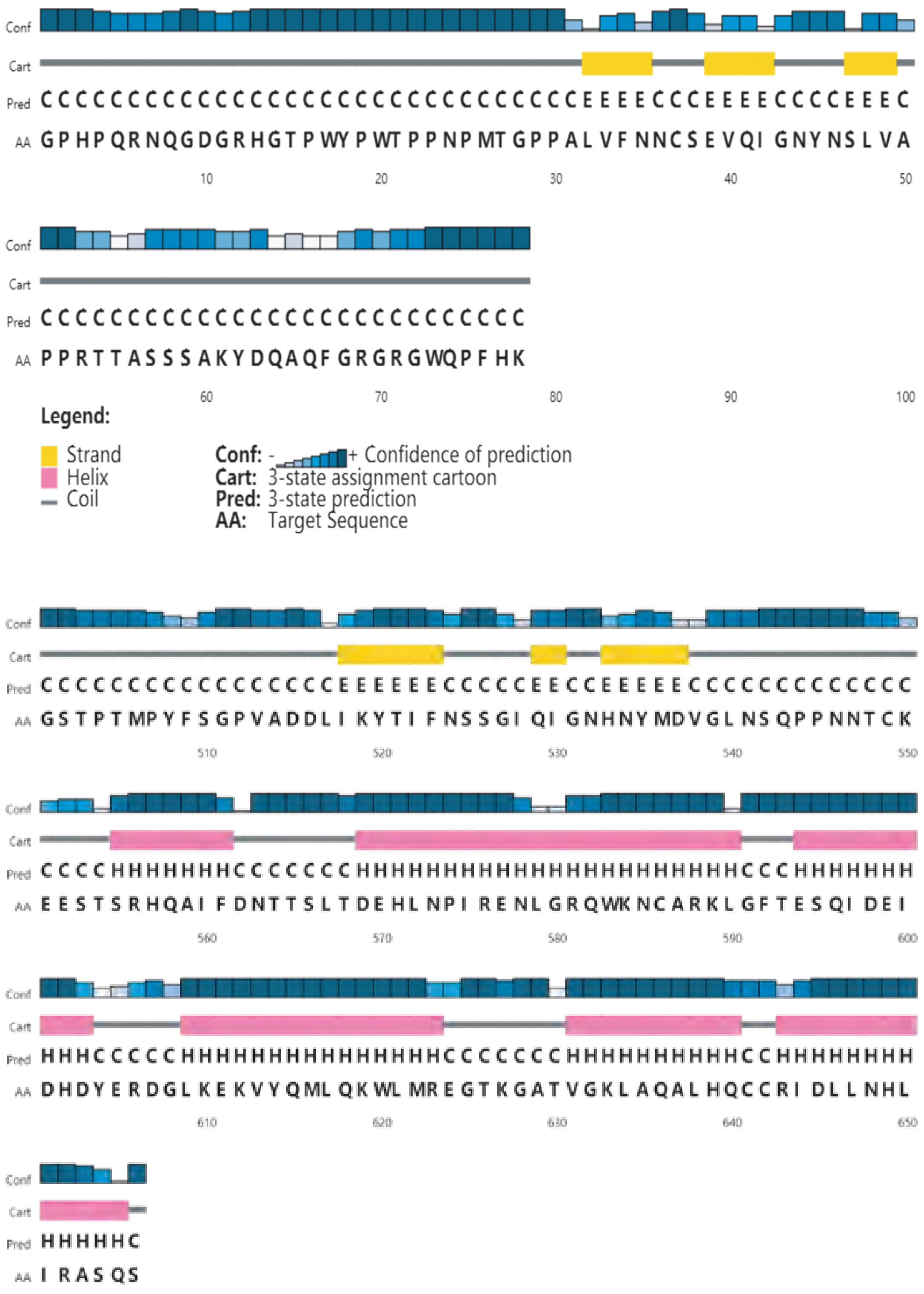

Supplementary Figure 1. T**he secondary structure prediction using PSIPRED indicating 3 β strands at RHIM domain. a** mouse RIPK3 sequence. **b** mouse RIPK1 sequence. The RHIM tetrad is the 2nd β-strand for both RIPK1 and RIPK3.

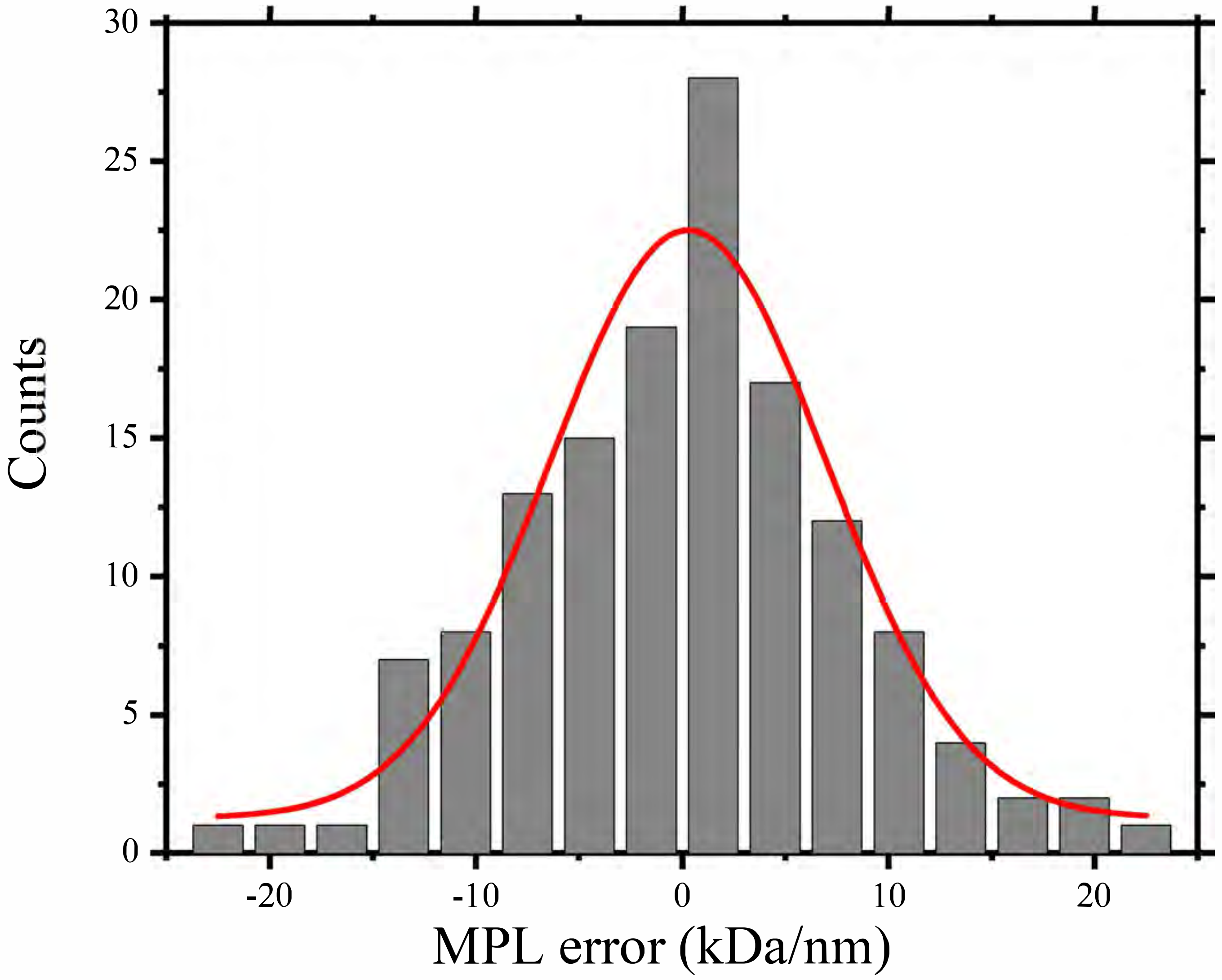

Supplementary Figure 2. **The image background analysis for MPL measurement of mouse RIPK3 fibrils.** The error were calculated by the same method described in the article[1]. We obtained 139 MPL error counts through measurement of the background intensity in the dark field images. Each reading was obtained for a rectangle with 60 nm×120 nm in size, same size as what was used in obtaining MPL values. Data was analyzed and plotted by Origin2018 with Gaussian fitting. The best-fit Gaussian function displays a width of 15.7 kDa/nm (full-width-at-the-half-height).

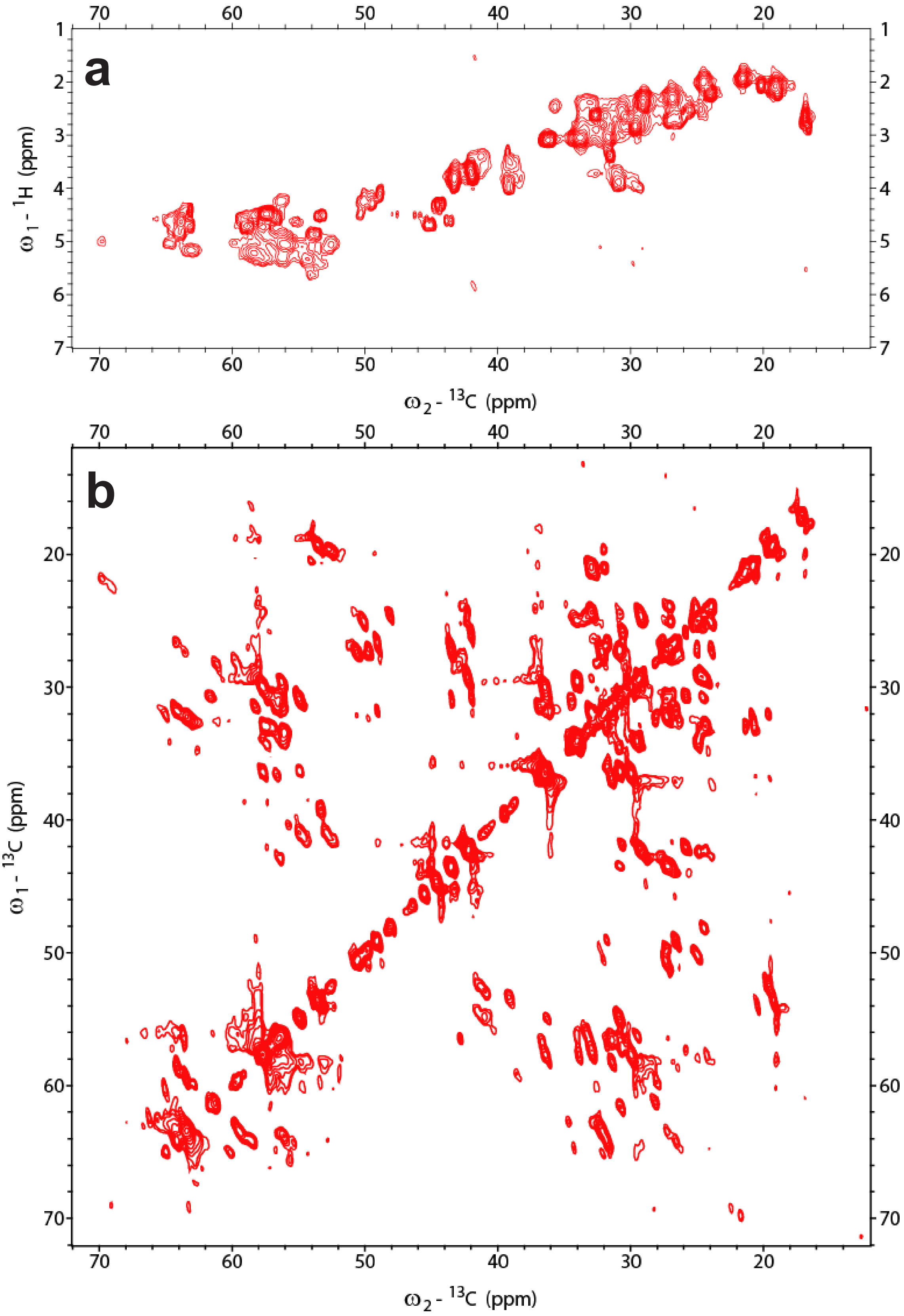

Supplementary Figure 3. **INEPT experiment to detect the flexible region of the fibril.** **a** INEPT spectrum of mouse RIPK3 fibrils. **b** INEPT-TOBSY spectrum of mouse RIPK3 fibrils.

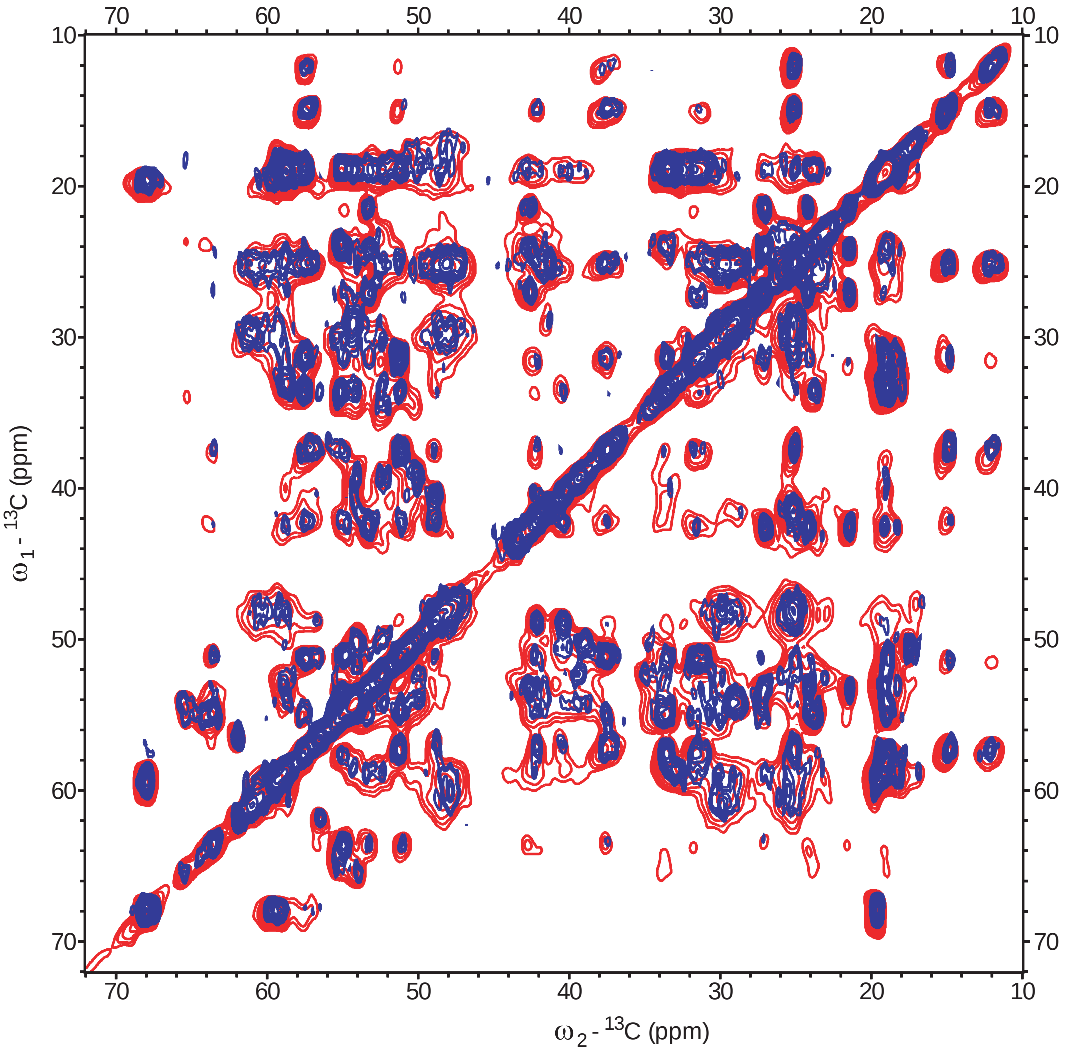

Supplementary Figure 4. **2D ^{13}C-^{13}C DARR spectra to distinct the intermolecular and intramolecular interactions.** 2D ^{13}C-^{13}C DARR spectrum of equimolar-mixed ^{13}C-labeled and ^{15}N-labeled mouse RIPK3 fibrils with a mixing time of 200 ms (blue) and the spectrum taken using the same parameters for uniformly labeled mouse RIPK3 fibril (red).

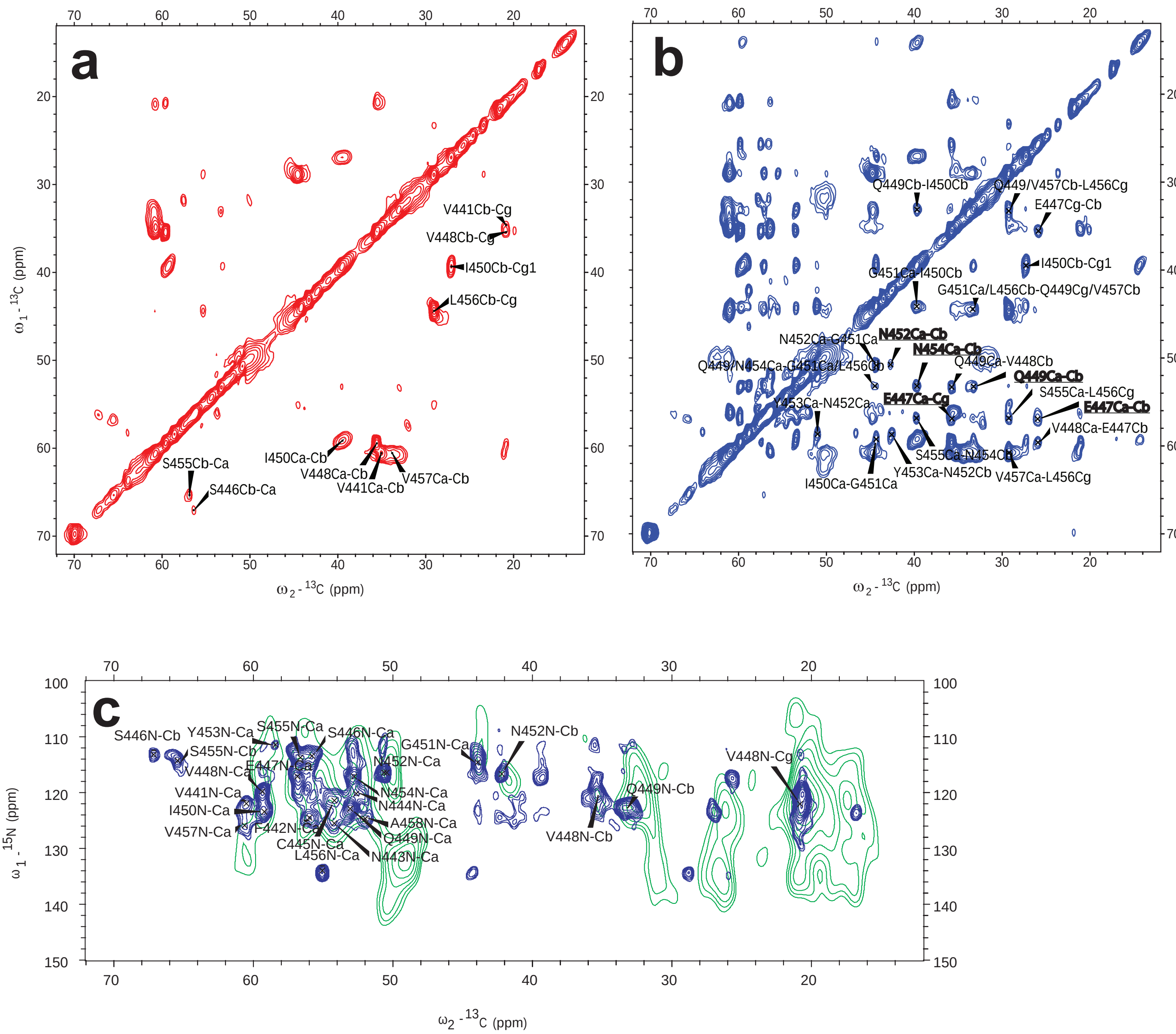

Supplementary Figure 5. **SSNMR confirms the parallel in-register β- sheet conformation of mouse RIPK3 fibril.** Comparison of 2D ^{13}C-^{13}C correlation spectra of sparsely ^{13}C-labeled mouse RIPK3 fibrils using [2-^{13}C]-labeled glycerol (**a**) 50 ms DARR mixing and (**b**) 500ms DARR mixing. **c** Comparison of ^{13}C-^{15}N spectra, Z-filtered TEDOR with 8.5 ms recoupling in green was carried out at 252K using fibrils with mixed labeling (^{13}C:^{15}N =1:1) while 2D NCaCX in blue was carried out at room temperature using uniformly [^{13}C,^{15}N]-labeled fibrils.

a

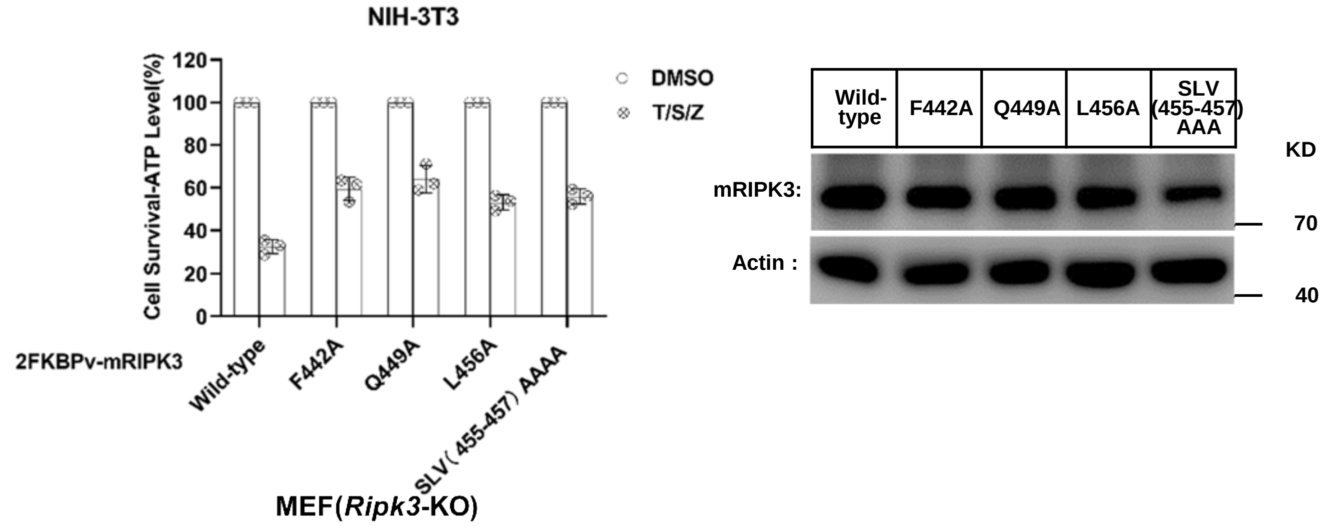

b

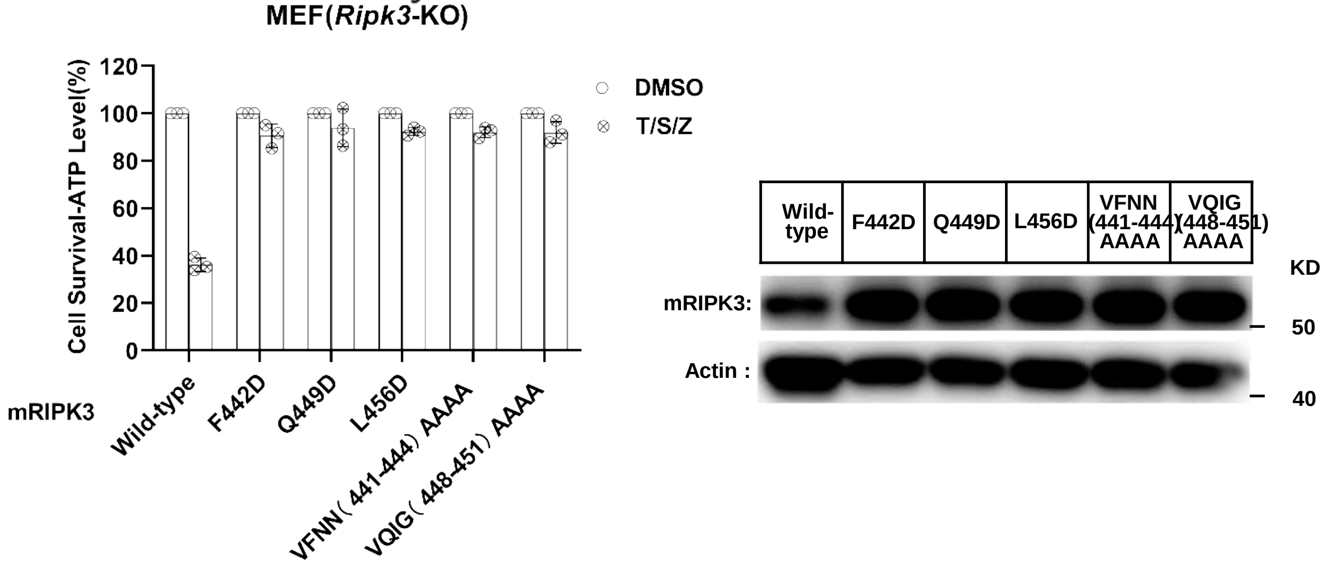

Supplementary Figure 6. **a** Mutation of Phe442, Gln449 or Leu456 in RIPK3 to Ala, or triple alanine mutations of Ser454/Leu456/Val455 in RIPK3 crippled the TNF-induced cell necroptosis. The NIH-3T3 cells with indicated lentivirus infection were treated T/S/Z for 10 h. The number of surviving cells was analyzed by measuring ATP levels (left). Data are presented as mean ± SD of n = 3 biologically independent replicates. Source data are provided as a Source Data file. The mouse RIPK3 (Sigma-Aldrich, PRS2283,1:3000) expression level was measured by western blot analysis (right). (B) The MEF(*Ripk3*-KO) cells stably expressing mouse wild and mutated RIPK3 protein by lentivirus infection were stimulated with T/S/Z for 10 hours. The number of surviving cells were analyzed by measuring ATP levels using Cell Titer-Glo kit (left). Data are presented as mean ± SD of n = 3 biologically independent replicates. Source data are provided as a Source Data file. The mouse RIPK3 (Sigma-Aldrich, PRS2283, 1:3000) expression level was measured by western blot analysis (right). All experiments were repeated three times. Uncropped blots in the Source Data file.

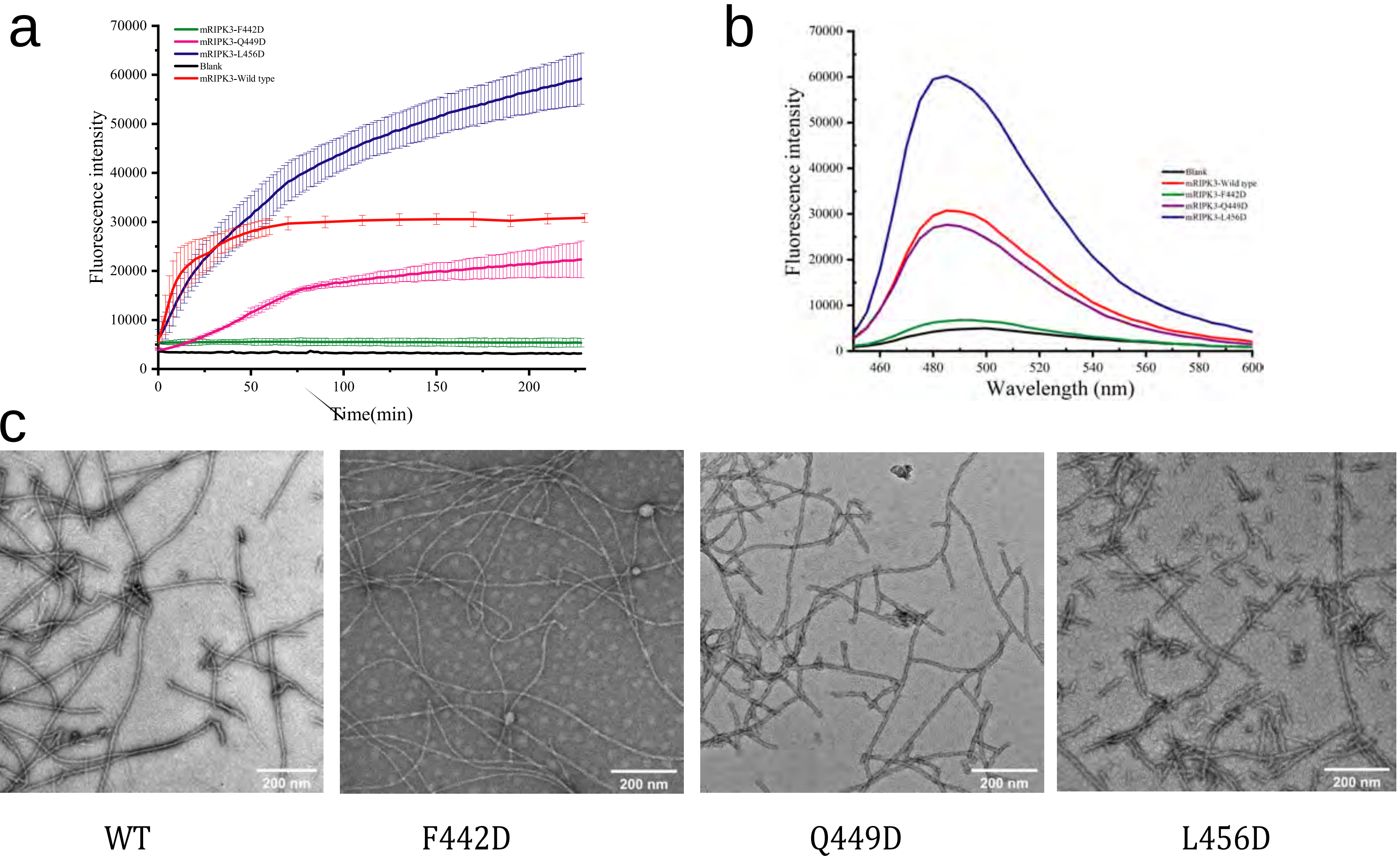

Supplementary Figure 7. **Characterization of wild type and mutant mouse RIPK3 fibrils using fluorescence and TEM. a** Fluorescence intensity increases during the fibril growth. In this experiment, 2mM protein stock solution in 6M GudHCl was diluted to a final concentration of 20μM in a 200μL volume using 10mM PB buffer (pH7.4). The fluorescence was measured immediately after the mixing. Data are presented as mean values +/- SD. The experiments were repeated three times. **b** The fluorescence profile of mouse RIPK3 wild-type and mutant fibrils. **c** TEM images of mouse RIPK3 wild-type and mutant fibrils. The experiments were repeated four times.

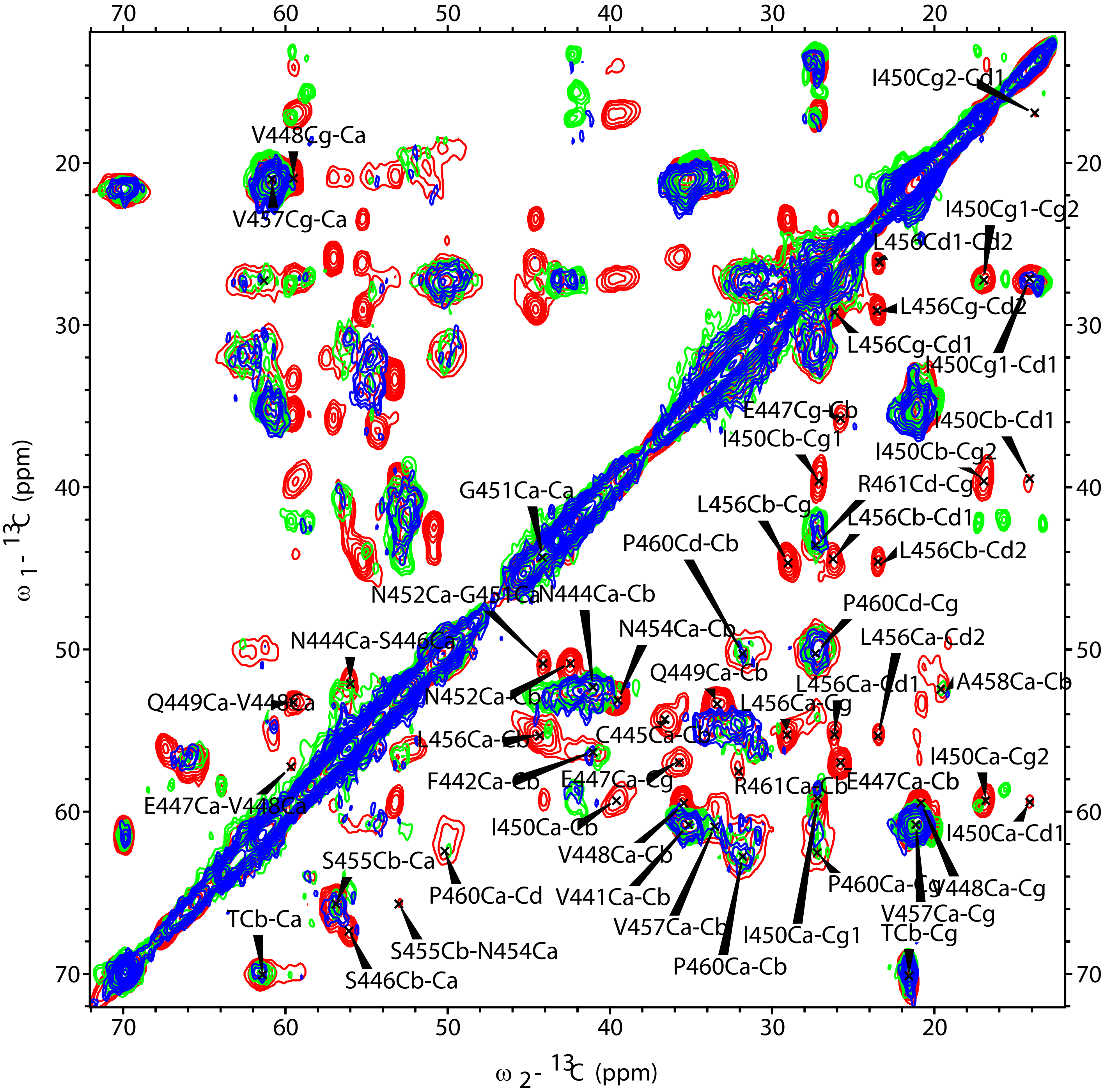

Supplementary Figure 8. **Comparison of 2D ^{13}C-^{13}C correlation spectra of uniformly ^{13}C-labeled mouse RIPK3 fibrils and its mutants.** Wild type (red), mutant Q449D (green), L456D (blue). The assignment in the spectra is for the wild type.

```
mouse RIPK1 TIFNSSGIQIGNHNYMDVGLN
            ...|:|.:||||:|.:.....
mouse RIPK3 VFNNCSEVQIGNYNSLVAPPR
```

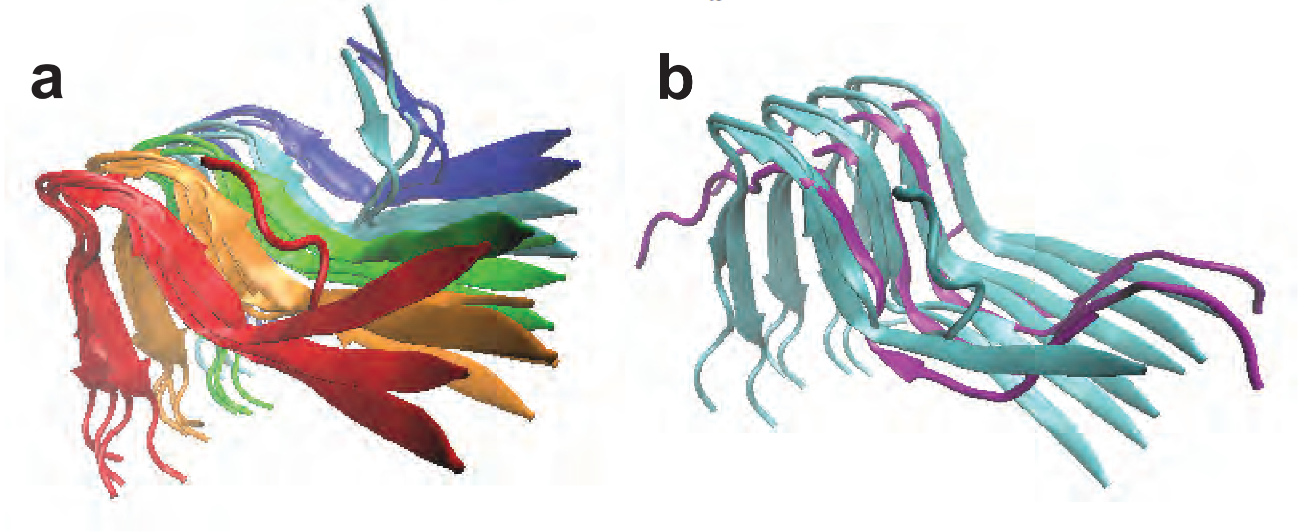

Supplementary Figure 9. **Molecular dynamics (MD) simulations on a hetero-amyloid model of mouse RIPK1/RIPK3 using a different sequence alignment shown in Figure 8. a** The best 4 structures of mouse RIPK1/RIPK3 hetero-amyloid after 50 ps MD, showing an opening the β-arches formed by 1st and 2nd β-strand. The sequence alignment for mouse RIPK1 and RIPK3 is shown on top. **b** The structure comparison between b (cyan, showing only the best 2 structures for clarity) and the human RIPK1/RIPK3 hetero-amyloid structure (purple, 5v7z.pdb).

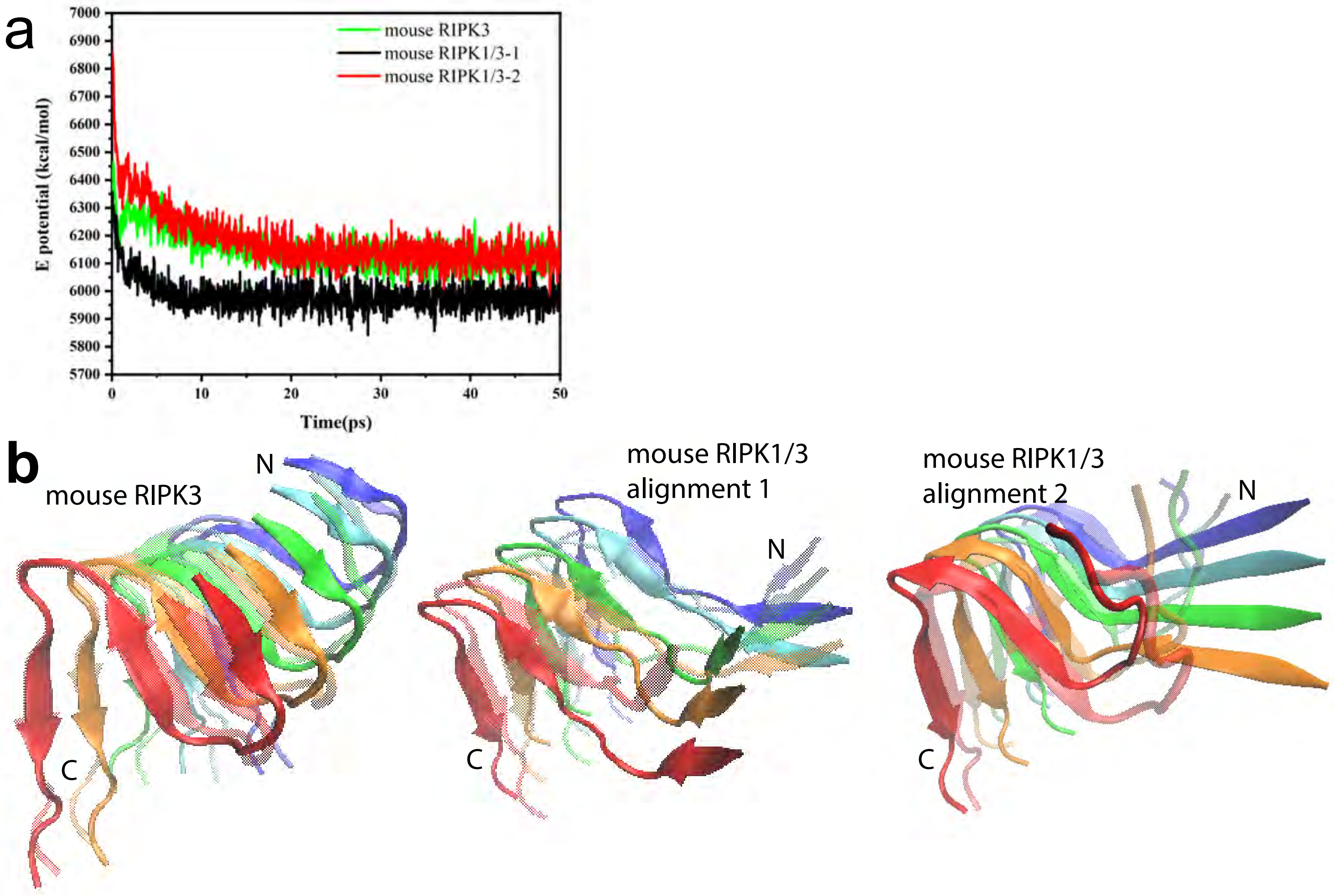

Supplementary Figure 10. **The simulation for RIPK3 amyloid fibrils. a** The energy including all potential energy terms (kcal/mol) changes along the 50 ps MD runs. The MD study of mouse RIPK3 fibril is in green; The MD runs for mouse RIPK1/RIPK3 fibril are shown in black and red for two different RIPK1 and RIPK3 alignments. The alignment 1 (black) corresponds to the alignment shown in figure 8c while the alignment 2 (red) corresponds to the alignment in Supplementary Figure9. All three are from the structures with the minimum energy among the 96 repeats. **b** The structural comparison between the best structure (solid color) and the worse structure (translucent) from the 96 repetitions of MD studies.

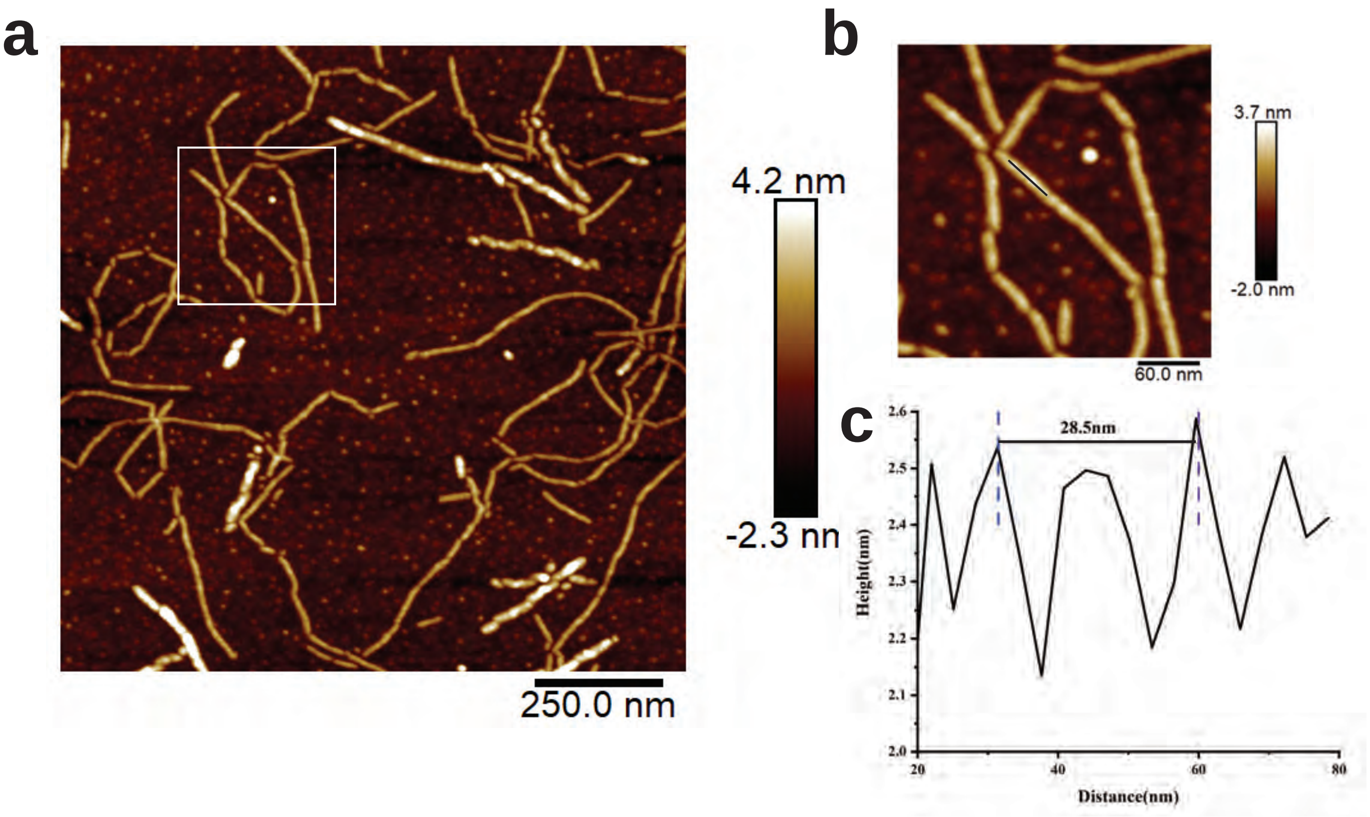

Supplementary Figure 11. **The AFM image for mouse RIPK3 fibrils. a** One AFM image of mouse RIPK3. **b** The expanded view of the box highlighted in A. **c** The periodic height changes along the line shown in b. The experiments were repeated three times.

Supplementary Table 1. **Experimental parameters in SSNMR for mRIPK3 fibril structure determination.**

sample	MAS (KHz)	Experiment	Recycle delay (s)	tppm (kHz)	Mixing time (ms)	Cross-polarization time (ms)	Number of scans	Total Time (h)	Temperature (K)
mRIPK3 (uniformly ^{13}C, ^{15}N labeled)	15	DARR	2	83.3	50	1.5	36	10.8	303
	15	DARR	2	83.3	200	1.5	36	11.5	303
	15	DARR	2	83.3	500	1.5	72	29.0	303
	15	2d-NCaCX	2	83.3	50	1.5, 5.5	256	7.2	303
	15	2d-NCoCX	2	83.3	50	1, 4.5	256	7.2	303
	15	3d-NCACX	2	83.3	50	1.5, 5.5	64	96	303
	15	3d-NCOCX	2	83.3	50	1.5, 5.5	64	59.5	303
	15	TEDOR	2	83.3	6.4	1.5	256	18.5	303
	15	TEDOR	2	83.3	8.5	1.5	256	18.5	303
mRIPK3 (2-^{13}C glycecol) and mRIPK3 (1,3-^{13}C glycecol)	15	DARR	2	100	50	1.5	48	14.3	303
	15	DARR	2	100	200	1.5	80	25.6	303
	15	DARR	2	100	500	1.5	96	34.8	303
	15	2d-NCaCX	2	100	50	1.5, 5.5	256	19.0	303
	15	2d-NCoCX	2	100	50	1.5, 4.5	256	19.0	303
	15	TEDOR	2	100	3.2, 6.4, 8.5	1.5	256	18.5	303
mRIPK3 (1:1 mixed $^{13}C/^{15}N$ labeled)	15	DARR	2	100	50	1.5	64	19.0	303
	15	DARR	2	100	200	1.5	64	20.5	303
	15	DARR	2	100	500	1.5	104	37.8	303
	15	TEDOR	2	83.3	6.4,8.5	1.5	256	18.5	252
	15	NhhC	2	83.3			512	23.3	252
	15	HXINEPT	2	83.3			128	20.4	303

Supplementary Table 2. **Chemical shift statistics from solid state NMR spectra of mouse RIPK3 fibrils.**

Residue	Chemical shifts (ppm)									
	N	CO	CA	CB	CG/CG1	CG2	CD/CD1	CD2	CE/CE1	CE2	CZ
V441	121.9	173.1	60.5	35.1	20.7	20.7					
F442	124.5	174.1	56	41.2	138.9		131.8	131.8	130.3	130.3	127.7
N443	125.6		54.2								
N444	120.5	173.1	51.9	40.8							
C445	120.9	178.1	54.1	36.2							
S446	113.1	173.5	55.9	67.1							
E447	117.1	173	56.9	25.5	35.5		181.5				
V448	120	174.5	59.4	35.3	20.7	20.7					
Q449	122.8	175.3	53	33.1	33.1		177				
I450	123.5	173.8	59.1	39.3	26.9	16.7	13.8				
G451	115	170.6	43.9								
N452	116.5	174.4	50.6	42.2			176.7				
Y453	111.4	175.3	58.4	35.2	131.6		133.5	133.5	117	117	156.5
N454	117	175.4	52.8	39.3			177				
S455	113.9	172.4	56.7	65.1							
L456	134	175	55	44.2	28.7		25.9	23.3			
V457	125.4		60.5	33.3	20.9	20.2					
A458	124.6		52.1	19.3							
P459			61	31.7	26.9		49.8				
P460			62.3	31.7	27		49.9				
R461	111.8		57.3	31.7	27.1		43.2				

Supplementary Table 3. **Structural restraints used in Xplor-NIH calculations.**

Xplor-NIH potential term											
CDIH						NOE					
torsion angles based on prediction by TALOS-N (all errors were expanded as described in the method)						parallel cross-β sheet (intermolecular alignment) (Å)			long-range contacts (residue crosspeaks)		
									unambi-guous	unambi-guous	Ambiguous (low ambiguity)
resid	Ψ	Φ	ΔΨ	ΔΦ	χ(Δχ)	C-C	H_n-O_{n-1}	N_n-O_{n-1}	TEDOR (4.5±2.5Å)	inter-residue DARR (5.5±1.5Å)	residues DARR (5.5±2.5Å)
V441									V441N-F442Ca, F442N-V441Cb, F442N-N443Ca, Q449Ne2-V448Ca, S455N-N454Cb, S455N-L456C, A457N-L456Cg, A457N-L456Cd2, N454Nd2-G451Ca, Q449Ne2-L456Cg	V441Cg#-G451Ca, S446Cb-V448Cb, I450Cg2-N452Ca, N452Cb-N454Ca, N452Ca-N454Ca, Q449Cd-L456Cb, Q449Cd-L456Cg, Y453Cb-S455Cb	V441/V448Cg#-I450/N454Cb, V441Cg-F442/N443//N452Cb, Q449Cb-I450/N454Cb, Q449Cg-I450/N454Cb, Q449Cd-E447/S455Ca, I450Cg2-V448/V441Cg, I450Cg2-Q449Cd/N452Cg, I450Cg2-F442/ N452Cb, I450Cg1/P459/P460Cg-F442Cg, G451C-Q449Cg/V457Cb, G451C-Q449/N454Ca, G451C-I450/N454Cb, N452Ca-I450/N454Cb, Y453Cg-E447/S455Ca, S455Cb-Q449Cg/V457Cb, S455Cb-V448/V457Cg, S455Cb-I450/Y453Ca, S446/S455-V448/V457Cg, L456Ca-I450/N454Cb, L456Cg-Q449Cg/V457Cb L456Cd2-Q449/N454Ca, L456Cd2- Q449/I450Cb,
F442	129.8	-106.4	35.0	35.0							
N443	135.6	-73.5	41.1	66.2							
N444		-115.4		73.6		4.75± 0.1	2.3±0.1	3.3±0.1			
C445							2.3±0.1	3.3±0.1			
S446	163.9	-118.4	35.0	70.2							
E447	41.7	56.4	35.0	35.0							
V448	145.1	-116.4	35.8	22.3		4.75± 0.1					
Q449	139.6	-116.2	37.0	46.8			2.3±0.1	3.3±0.1			
I450	124.5	-114.3	35.0	35.7	-58.1 (6.6)						
G451	168.8		52.2				2.3±0.1	3.3±0.1			
N452	157.2	-101.9	35.0	75.1							
Y453	28.5	63.6	39.1	35.0	-52.9 (12.3)						
N454		-77.2		36.0	-69.4 (11.8)						
S455	128.0	-115.4	53.2	55.4		4.75± 0.05					
L456	129.6	-103.3	35.0	42.5	177.9 (8.0)		2.3±0.1	3.3±0.1			
V457	134.4		48.4		-177.6 (17.0)						
A458	146.9	-68.8	47.3	45.4							
P459	147.4	-61.4	35.0	35.0							
P460	151.1	-64.4	35.0	35.0							
R461											

Supplementary Table 4. **Structure statistics for mouse RIPK3**

constraints	number
dihedral angles	32
chi angles	5
unambiguous intramolecular residues contacts	97 (10 non-sequential)
ambiguous intramolecular residues contacts	22
intermolecular constraints	parallel beta-sheet
MolProbity Clashscore	6
MolProbity Ramachandran outliers	0
MolProbity sidechain conformer outliers	2.1%
backbone RMSD(Å)	0.28

Supplementary Table 5. **The primers used in the paper for protein expression.**

primer	characters	source
mripk3(409- 486)	Forward:5’GGAATTCCA/TATG(N*deI*)CATCATCATCATCATCATGG TCCTCACCCCCAAAGG3’	Sangon Biotech
mripk3(409- 486)	Reverse:5’CCGC/TCGAG(X*hoI*)CTACTTGTGGA AGGGCTGC3’	Sangon Biotech
F442D mripk3	Forward: CTTCAGAACAGTTGTTGTCGACGAGAGCCGGTGGC	Sangon Biotech
F442D mripk3	Reverse: GCCACCGGCTCTCGTCGACAACAACTGTTCTGAAG	Sangon Biotech
Q449D mripk3	Forward: GGAGTTGTAGTTCCCAATATCCACTTCAGAACAG	Sangon Biotech
Q449D mripk3	Reverse: CTGTTCTGAAGTGGATATTGGGAACTACAACTCC	Sangon Biotech
L456D mripk3	Forward: TCTTGGTGGTGCTACATCGGAGTTGTAGTTCCCAATCTG	Sangon Biotech
L456D mripk3	Reverse: CAGATTGGGAACTACAACTCCGATGTAGCACCACCAAGA	Sangon Biotech
VFNN(441-444) AAAA mripk3	Forward:CAGGGCCACCGGCTCTCGCTGCAGCAGCTTGTTCTGAAG TGCAGATTGGG	Sangon Biotech
VFNN(441-444) AAAA mripk3	Reverse:CCCAATCTGCACTTCAGAACAAGCTGCTGCAGCGAGAGC CGGTGGCCCTG	Sangon Biotech
VQIG(448-451) AAAA mripk3	Forward:CAACAACTGTTCTGAAGCTGCAGCAGCTAACTACAACTCC TTGG	Sangon Biotech
VQIG(448-451) AAAA mripk3	Reverse:CCAAGGAGTTGTAGTTAGCTGCTGCAGCTTCAGAACAGTT GTTG	Sangon Biotech
SLV(455-457)AAA mripk3	Forward:CAGATTGGGAACTACAACGCTGCAGCAGCTCCACCAAGAA CTACTGCC	Sangon Biotech
SLV(455-457)AAA mripk3	Reverse:GGCAGTAGTTCTTGGTGGAGCTGCTGCAGCGTTGTAGTTC CCAATCTG	Sangon Biotech

Supplementary Reference:

1. Murray, D. T. et al. Structure of FUS Protein Fibrils and Its Relevance to Self-Assembly and Phase Separation of Low-Complexity Domains. Cell 171, 615-627 e616, doi:10.1016/j.cell.2017.08.048 (2017)
