# Supplementary material for: The amyloid structure of mouse RIPK3 (receptor interacting protein kinase 3) in cell necroptosis
Source: Nat Commun. 2021 Mar 12;12:1627. doi: 10.1038/s41467-021-21881-2 (PMC7955032; doi:10.1038/s41467-021-21881-2)
Supplement: Supplementary file 3 — Reporting Summary [file 41467_2021_21881_MOESM3_ESM.pdf]

nature research

Corresponding author(s): Jun-xia, Lu; Hua-yi, Wang

Last updated by author(s): Feb 1, 2021

Reporting Summary

Nature Research wishes to improve the reproducibility of the work that we publish. This form provides structure for consistency and transparency in reporting. For further information on Nature Research policies, see our Editorial Policies and the Editorial Policy Checklist.

Statistics

For all statistical analyses, confirm that the following items are present in the figure legend, table legend, main text, or Methods section.

n/a	Confirmed	
☐	☒	The exact sample size (n) for each experimental group/condition, given as a discrete number and unit of measurement
☐	☒	A statement on whether measurements were taken from distinct samples or whether the same sample was measured repeatedly
☒	☐	The statistical test(s) used AND whether they are one- or two-sided<br>*Only common tests should be described solely by name; describe more complex techniques in the Methods section.*
☒	☐	A description of all covariates tested
☒	☐	A description of any assumptions or corrections, such as tests of normality and adjustment for multiple comparisons
☐	☒	A full description of the statistical parameters including central tendency (e.g. means) or other basic estimates (e.g. regression coefficient) AND variation (e.g. standard deviation) or associated estimates of uncertainty (e.g. confidence intervals)
☒	☐	For null hypothesis testing, the test statistic (e.g. F, t, r) with confidence intervals, effect sizes, degrees of freedom and P value noted<br>*Give P values as exact values whenever suitable.*
☒	☐	For Bayesian analysis, information on the choice of priors and Markov chain Monte Carlo settings
☒	☐	For hierarchical and complex designs, identification of the appropriate level for tests and full reporting of outcomes
☒	☐	Estimates of effect sizes (e.g. Cohen's d, Pearson's r), indicating how they were calculated

Our web collection on statistics for biologists contains articles on many of the points above.

Software and code

Policy information about availability of computer code

Data collection	xplor-NIH(version 2.48, including the PosDiffPot, TorsionDB module), Topspin 4.02(bruker), NanoScope Analysis 1.8, APEX3 V2018.7-2, Talas L120 C (6.15.3), TIA 5.0.0
Data analysis	Image J (NIH. 1.52v), Sparky, nmrPipe, VMD, Origin2018, TALOS-N, PSIPRED, WALTZ, Clustalw2,adxv.x86_64RHEL6 program (Scripps Research Institute, La Jolla, CA, USA)

For manuscripts utilizing custom algorithms or software that are central to the research but not yet described in published literature, software must be made available to editors and reviewers. We strongly encourage code deposition in a community repository (e.g. GitHub). See the Nature Research guidelines for submitting code & software for further information.

Data

Policy information about availability of data

All manuscripts must include a data availability statement. This statement should provide the following information, where applicable:

- Accession codes, unique identifiers, or web links for publicly available datasets
- A list of figures that have associated raw data
- A description of any restrictions on data availability

Source data are provided with this paper. The solid-state NMR structure of mouse RIPK3 was deposited as PDB ID: 6JPD [https://www.rcsb.org/structure/6JPD] and NMR chemical shifts were deposited into BMRB: 36243 [https://bmrb.io/data_library/summary/index.php?bmrbId=36243].

Field-specific reporting

Please select the one below that is the best fit for your research. If you are not sure, read the appropriate sections before making your selection.

[x] Life sciences [ ] Behavioural & social sciences [ ] Ecological, evolutionary & environmental sciences

For a reference copy of the document with all sections, see nature.com/documents/nr-reporting-summary-flat.pdf

Life sciences study design

All studies must disclose on these points even when the disclosure is negative.

Sample size	Sample size was determined based on standards for experimental cell biology , attempting to have a minimum of N = 3 biological replicates with sufficient reproducibility
Data exclusions	No data exclusions were done. All experimental data were taken into the consideration for the final conclusion.
Replication	Cell-based assay was repeated three times. AFM was repeated three times and BT-TEM was repeated 10 times. NMR studies were carried out on three types of samples with at least 4 repeated. All the repeated experiment indicated similar results. All findings were replicated successfully.
Randomization	Samples were allocated randomly for culture and analysis.
Blinding	For our study, blinding is not necessary since our readouts such as % of cell death and protein levels are not largely subjective.

Reporting for specific materials, systems and methods

We require information from authors about some types of materials, experimental systems and methods used in many studies. Here, indicate whether each material, system or method listed is relevant to your study. If you are not sure if a list item applies to your research, read the appropriate section before selecting a response.

Materials & experimental systems

n/a	Involved in the study	
[ ]	[x]	Antibodies
[ ]	[x]	Eukaryotic cell lines
[x]	[ ]	Palaeontology and archaeology
[x]	[ ]	Animals and other organisms
[x]	[ ]	Human research participants
[x]	[ ]	Clinical data
[x]	[ ]	Dual use research of concern

Methods

n/a	Involved in the study	
[x]	[ ]	ChIP-seq
[x]	[ ]	Flow cytometry
[x]	[ ]	MRI-based neuroimaging

Antibodies

Antibodies used	anti-mRIPK3 (Sigma-Aldrich, PRS2283, 1:3000); anti-RIPK1 (Cell Signaling Technology, D94C12, 1:1000); Anti-actin(MBL, PM053-7, 1:10000)
Validation	All primary antibodies used were validated for their use in western blot and/or immunoprecipiation procedures as indicated according to manufacturer's website or literature, including:<br>anti-RIPK1 (Cell Signaling Technology, D94C12, 1:1000) has been validated for Western-blots of human and mouse samples by previous publications (e.g .PMID:28506461,PMID: 26642243).<br>anti-mRIPK3 (Sigma-Aldrich, PRS2283) has been validated for Western-blots of mouse samples by previous publications (e.g .PMID: 31817643).

Eukaryotic cell lines

Policy information about cell lines

Cell line source(s)	HEK293T cells(ATCC); NIH-3T3 (ATCC); Ripk3-knockout MEF cells were generated from Ripk3-knockout genotype of mice.
Authentication	no further validation
Mycoplasma contamination	All cell lines were tested negative for mycoplasma contamination.

Commonly misidentified lines<br>(See ICLAC register)	No
